# Multiomics analysis of COL12A1 as a promising prognostic biomarker for immune-related treatment of gastric cancer

**DOI:** 10.1007/s12672-025-03405-2

**Published:** 2025-10-14

**Authors:** Jin Shi, Chenggong Zhang, Zihao Gao, Fan Ding, Dezhu Dai, Xu Wu, Xiao Han, Liang Shi, Xudong Song, Guoquan Tao

**Affiliations:** 1 Department of Gastrointestinal Surgery, The Affiliated Huaian No. 1 People’s Hospital of Nanjing Medical University, Huaian, 223300 Jiangsu China; 2 Department of Pediatric Surgery, University Children’s Hospital Basel, Basel, 4031 Switzerland; 3 Department of Clinical Research, University of Basel, Basel, 4031 Switzerland; 4 Department of Vascular, Huaian Hospital affiliated with Xuzhou Medical University, Huaian, 223300 Jiangsu China

**Keywords:** COL12A1, Gastric cancer, Immune infiltration, Methylation, Extracellular matrix

## Abstract

COL12A1, a collagen type XII alpha 1 chain, has an instrumental role in the extracellular matrix (ECM), but its effect on gastric cancer (GC) and clinical significance remain unclear. In this study, co-expressed differentially expressed genes (co-DEGs) were identified using Venn diagrams based on datasets from The Cancer Genome Atlas (TCGA) and the Gene Expression Omnibus (GEO) for gastric cancer RNA sequencing. Intersections between co-DEGs and TCGA prognosis-related genes were used to pinpoint genes that were differentially associated with prognosis. COL12A1 emerged as a key candidate gene. We analyzed COL12A1 expression in human GC tissues and explored its association with various clinicopathological parameters. Survival analysis, including overall survival (OS) and disease-free survival (DFS), was conducted using TCGA data, and a prognostic nomogram was constructed. The relative protein-encoding gene expression was assessed via immunohistochemistry (IHC) analysis of four downloaded datasets, along with PCR experiments. COL12A1-related DEGs were further identified using the LinkedOmics database, followed by Gene Ontology (GO) and Kyoto Encyclopedia of Genes and Genomes (KEGG) enrichment analyses. Multiple databases were analysed for the relationship between COL12A1 and methylation. Immune cell infiltration was evaluated through CIBERSORT and TIMER databases, revealing a strong correlation between COL12A1 expression and immune cell presence in the tumor microenvironment. Single-cell expression analysis using TISCH provided further insights into the cellular context of COL12A1 expression. TISCH was used to analyse the single-cell expression of COL12A1. Finally, this work examined the correlation of COL12A1 with drug sensitivity via the RNAactDrug database. COL12A1 overexpression in gastric cancer was detected and foundto be closely related to clinicopathological features. GO/KEGG functional enrichment analysis revealed that COL12A1 was associated with pathways such as extracellular structure organization, extracellular matrix organization and the collagen-containing extracellular matrix. Analysis of several databases revealed that COL12A1 was associated with gene mutation and methylation. Additionally, as revealed by CIBERSORT profiling and TIMER database analysis, COL12A1 expression is related to immune cell infiltration. Correlation analysis via the RNAactDrug website revealed that COL12A1 is associated with drug sensitivity. In conclusion, COL12A1 is significantly overexpressed in gastric cancer and is associated with tumor progression, immune infiltration, and drug sensitivity. These findings suggest that COL12A1 may serve as a potential diagnostic and therapeutic biomarker for gastric cancer.

**Supplementary Information:**

The online version contains supplementary material available at 10.1007/s12672-025-03405-2.

## Introduction

Gastric cancer (GC) is a major global health challenge, ranking fifth in incidence and third in cancer-related mortality worldwide ( [1]– [2]). The most commonly diagnosed type of GC is stomach adenocarcinoma, a life-threatening malignancy with a substantial global burden. Although several novel therapies for gastric cancer (GC), including surgery, radiotherapy, chemotherapy, immunotherapy and targeted therapy, have been developed in recent years [3], the mortality and relapse rates remain high, especially in advanced GC, with a 5-year survival rate as low as 10% [4]. Therefore, it is crucial to investigate the molecular mechanisms underlying gastric carcinogenesis, identify key genes involved in tumor progression, and assess their potential as early diagnostic markers and therapeutic targets.

Tumor progression in the stomach is strongly influenced by changes in the tumor microenvironment (TME), particularly those involving the extracellular matrix (ECM). The deposition and remodeling of the ECM surrounding tumor cells can disrupt cell polarity and weaken cell adhesion, thereby facilitating tumor growth, invasion, and metastasis. As the primary structural component of the ECM, collagen plays a pivotal role in modulating the TME in gastric cancer [5]. Previous studies have shown that several collagen genes, including COL3A1, COL4A1, and COL11A1, are significantly associated with GC progression and may serve as novel biomarkers for diagnosis and prognosis [6–8]. Thus, identifying molecules that regulate collagen deposition or synthesis within the TME could enhance early diagnostic accuracy and improve clinical outcomes in GC.

Among the various ECM-associated genes, COL12A1, a member of the fibril-associated collagens with interrupted triple helices (FACIT) family, has emerged as a particularly intriguing candidate in gastric cancer. Traditionally recognized for its structural role, COL12A1 has recently been implicated in cancer progression. Elevated expression of COL12A1 has been correlated with poor prognosis and increased metastatic potential in GC patients (reference). Moreover, its overexpression has been documented in several other cancer types, suggesting a broader role in tumorigenesis (reference needed). Despite these associations, the precise biological mechanisms by which COL12A1 contributes to gastric cancer remain poorly understood. Understanding these mechanisms may offer new opportunities for the development of diagnostic biomarkers and targeted therapies directed at the tumor microenvironment.

In this study, we aimed to investigate the potential role of COL12A1 in the progression of gastric cancer. We integrated data from The Cancer Genome Atlas (TCGA) and three Gene Expression Omnibus (GEO) datasets to comprehensively assess COL12A1 expression and its clinical relevance in GC. TCGA data were used to evaluate correlations between COL12A1 expression and clinicopathological parameters, patient prognosis, and immune cell infiltration within the TME. In parallel, multidimensional analyses of the GEO datasets were performed to explore potential regulatory networks and functional pathways associated with COL12A1. Finally, the bioinformatic findings were validated througha series of in vitro and invivo experiments to confirm the role of COL12A1 in GC progression. Collectively, our findings suggest that COL12A1 may act as a tumor-promoting gene in gastric cancer, potentially by modulating immune dynamics within the tumor microenvironment.

## Results

### Selection of differential genes

**Fig. 1:**
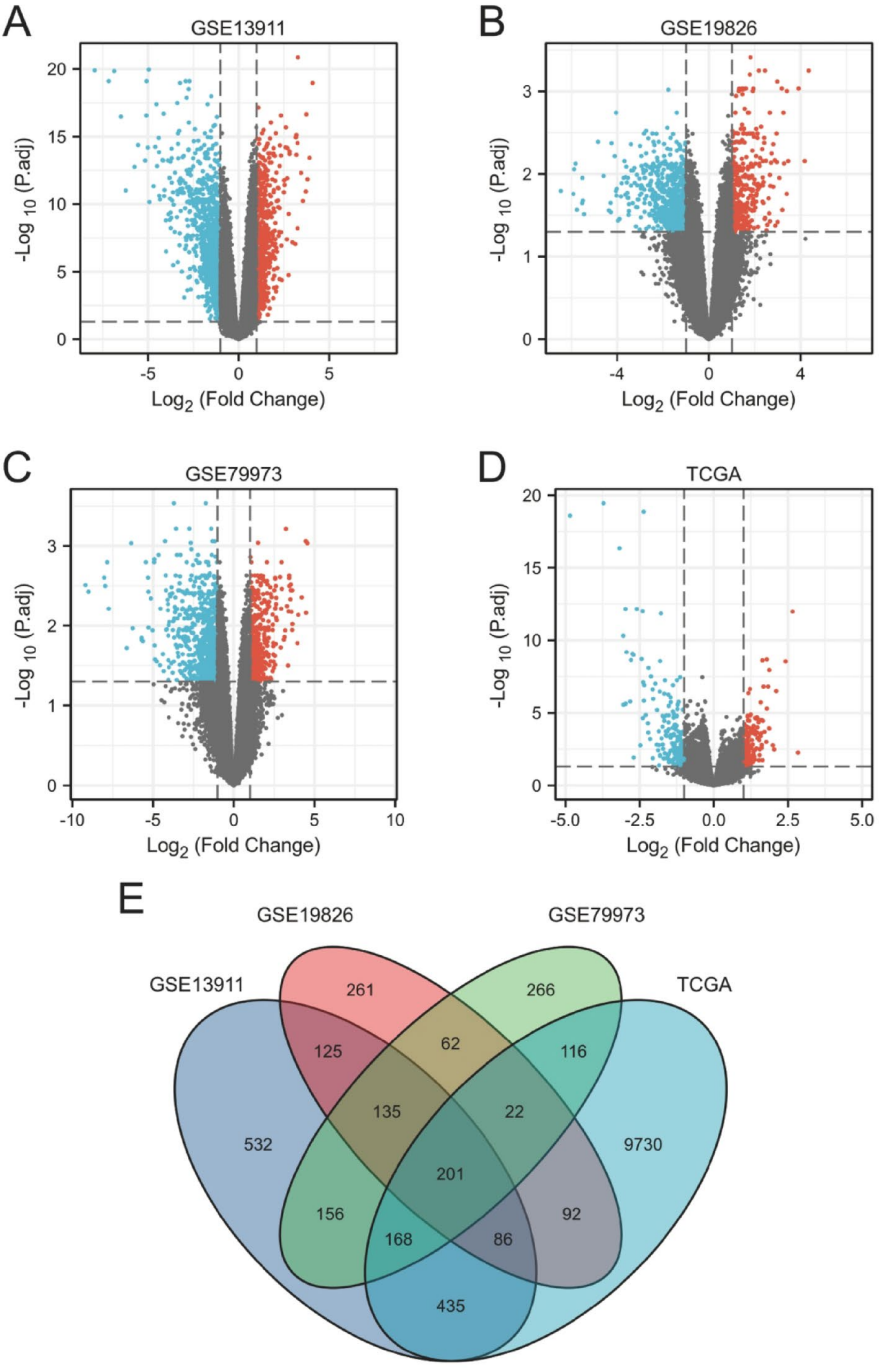
Differentially expressed genes (DEGs) between gastric cancer and normal tissues. Volcano plots of DEGs in the (**A**) GSE13911, (**B**) GSE19826, (**C**) GSE79973 and (**D**) TCGA datasets. (**E**) Venn diagram distribution of DEGs

Three gastric cancer-related datasets (GSE13911, GSE19826, and GSE79973) from the GEO database, along with STAD data from TCGA, were analysed. Differential gene expression between gastric cancer and normal tissues was assessed using the “limma” package in R. DEGs were defined using the criteria |logFC| >1 and adjusted p-value < 0.05. Volcano plots were generated to visualize the upregulated (red) and downregulated (green) genes (Fig. 1A–D). The intersecting DEGs from the three GEO datasets and the TCGA dataset yielded 201 shared DEGs, including 65 that were upregulated (Fig. 1E).

### Screening for prognosis-associated genes

**Fig. 2:**
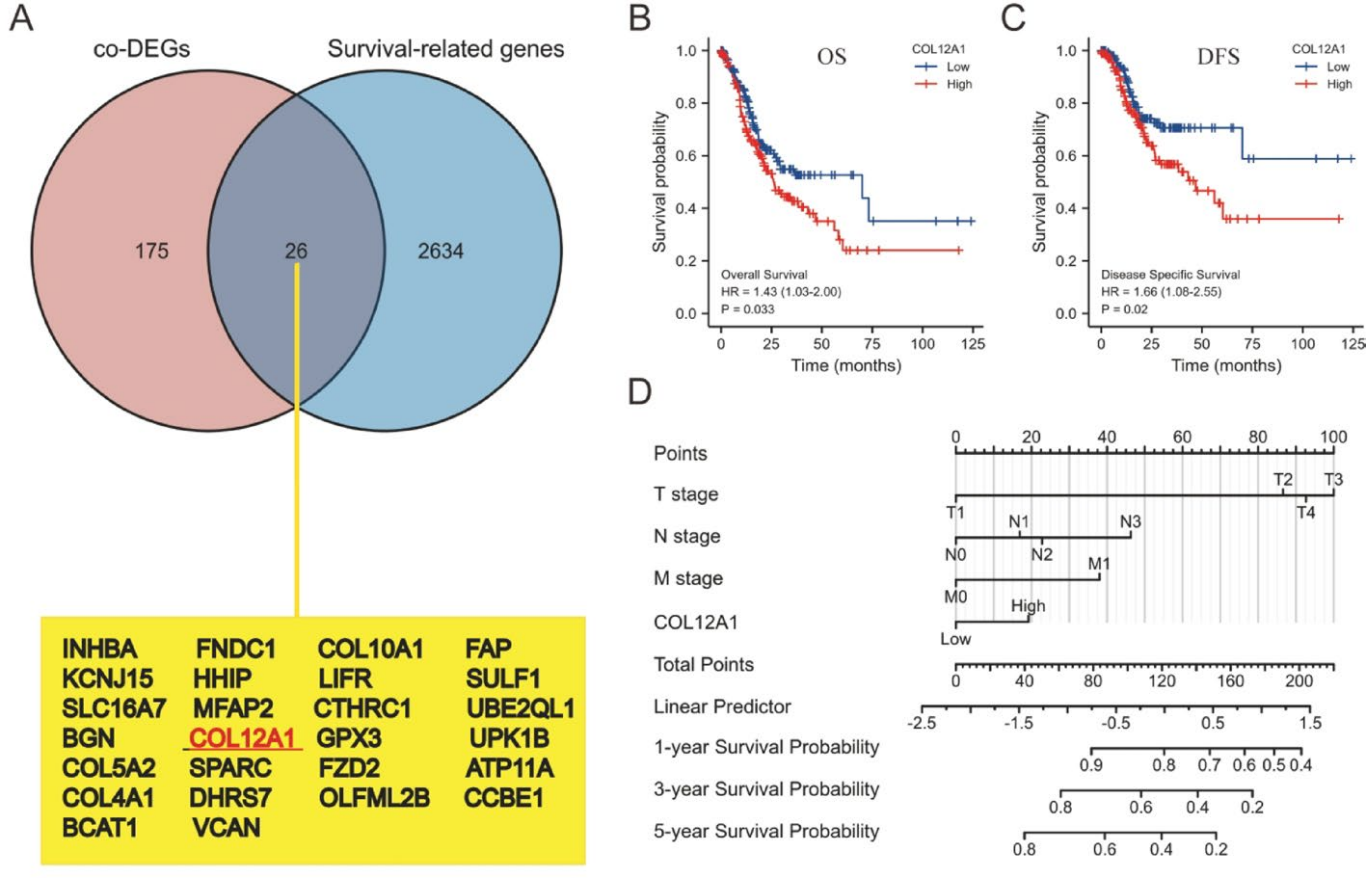
COL12A1 expression is associated with the prognosis of gastric cancer patients. (**A**) Venn diagram of the DEGs associated with prognosis and the 26 overlapping genes. The survival curves of (**B**) OS and (**C**) DSS in patients with high and low expression of COL12A1 in STAD were compared. (**D**) Nomogram survival prediction chart for predicting the 1-, 3-, and 5-year overall survival rates

To identify prognostic biomarkers, we analysed RNA-seq data from the TCGA-STAD project and performed Cox regression on the DEGs. A Venn diagram revealed 26 overlapping genes with prognostic relevance (Fig. 2A). Among these, COL12A1 was significantly upregulated in GC and strongly associated with poor prognosis. Kaplan–Meier survival analysis demonstrated that patients with high COL12A1 expression had significantly shorter overall survival (OS, *p* = 0.033; Fig. 2B) and disease-specific survival (DSS, *p* = 0.02; Fig. 2C). A prognostic nomogram combining COL12A1 expression with clinical variables was constructed to predict 1-, 3-, and 5-year survival probabilities [11]. The model suggested that high COL12A1 expression is associated with a worse prognosis (Fig. 2D). Therefore, COL12A1 was selected for further analysis.

### Correlation of COL12A1 expression with clinicopathological features

We used chi-square tests to evaluate the association between COL12A1 expression levels and clinical features. Significant correlations were observed between COL12A1 overexpression and tumor T stage as well as histological grade (*p* < 0.05, Table 1), indicating a relationship between COL12A1 expression and tumor progression. Notably, patients with high COL12A1 expression tended to present with more advanced T stages and poorer histological differentiation.

**Table 1:** Correlations of COL12A1 levels with clinicopathological characteristics (TCGA)

Characteristic	Low expression of COL12A1	High expression of COL12A1	*p*
n	187	188	
T stage, n (%)			**0.005**
T1	16 (4.4%)	3 (0.8%)	
T2	39 (10.6%)	41 (11.2%)	
T3	90 (24.5%)	78 (21.3%)	
T4	41 (11.2%)	59 (16.1%)	
N stage, n (%)			0.515
N0	53 (14.8%)	58 (16.2%)	
N1	55 (15.4%)	42 (11.8%)	
N2	35 (9.8%)	40 (11.2%)	
N3	38 (10.6%)	36 (10.1%)	
M stage, n (%)			0.382
M0	169 (47.6%)	161 (45.4%)	
M1	10 (2.8%)	15 (4.2%)	
Pathologic stage, n (%)			0.828
Stage I	29 (8.2%)	24 (6.8%)	
Stage II	56 (15.9%)	55 (15.6%)	
Stage III	75 (21.3%)	75 (21.3%)	
Stage IV	17 (4.8%)	21 (6%)	
Histological type, n (%)			**0.023**
Diffuse Type	26 (7%)	37 (9.9%)	
Mucinous Type	5 (1.3%)	14 (3.7%)	
Not Otherwise, Specified	107 (28.6%)	100 (26.7%)	
Papillary Type	3 (0.8%)	2 (0.5%)	
Signet Ring Type	3 (0.8%)	8 (2.1%)	
Tubular Type	43 (11.5%)	26 (7%)	
Histologic grade, n (%)			0.588
G1	5 (1.4%)	5 (1.4%)	
G2	74 (20.2%)	63 (17.2%)	
G3	106 (29%)	113 (30.9%)	

### Pathway enrichment analysis

**Fig. 3:**
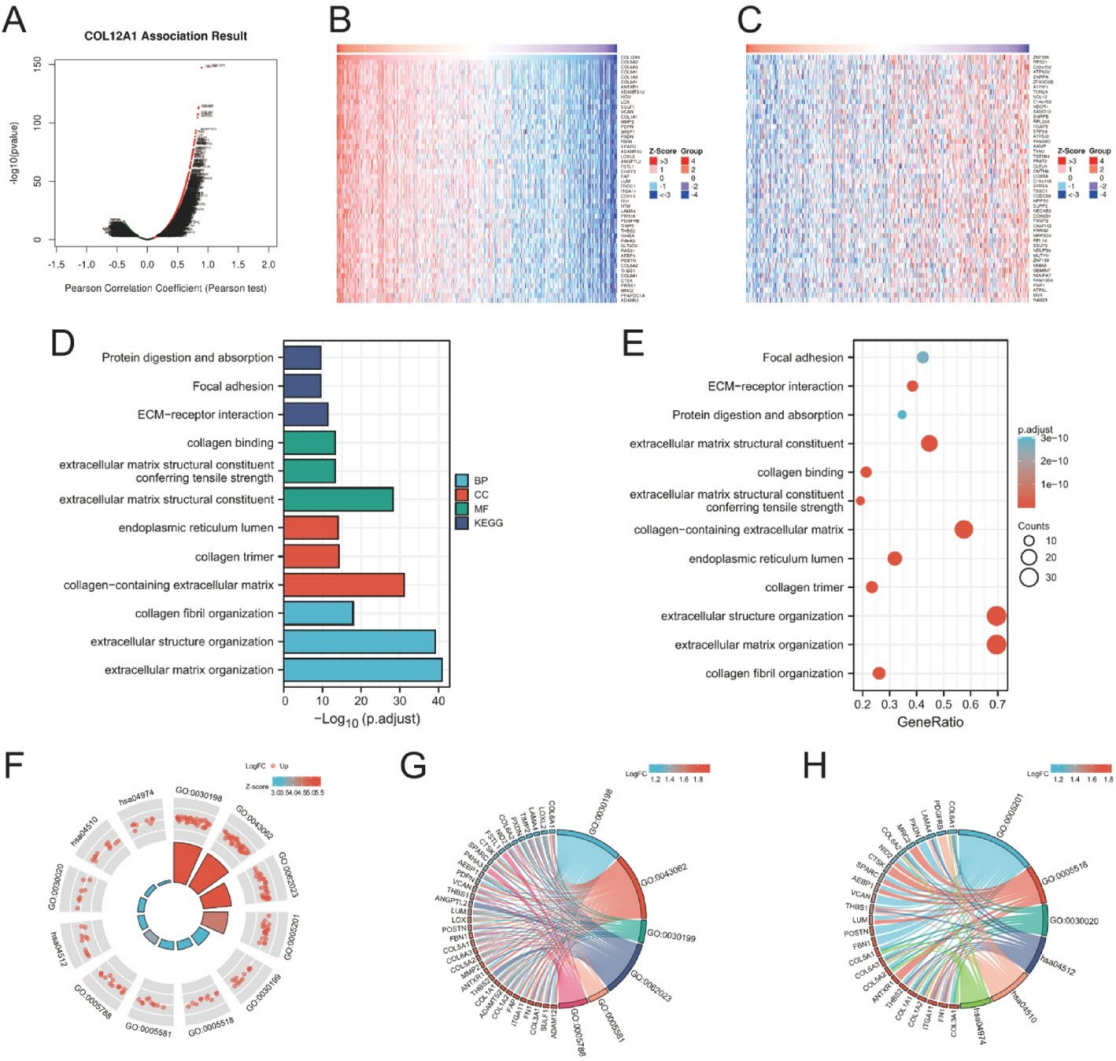
Enrichment analysis of the COL12A1 functional network in STAD. (**A**) Pearson’s chi-square test of genes identified in the STAD cohort that are highly correlated with COL12A1. (**B**) Heatmap showing the top 50 genes positively associated with COL12A1 in the STAD cohort. (**C**) Heatmap showing the top 50 genes negatively associated with COL12A1 in the STAD cohort. (**D**,** E**) Gene Ontology (GO) enrichment of genes associated with GLUT1 and Kyoto Encyclopedia of Genes and Genomes (KEGG) terms. (**F**) Corresponding GO and KEGG visual string diagrams. (**G**) Corresponding GO visualization circle diagrams. (**H**) Corresponding KEGG visualization circle diagrams

To explore the biological functions of COL12A1 in STAD, we performed coexpression analysis using the LinkedOmics database [12]. A total of 5087 genes were positively correlated and 4252 genes negatively correlated with COL12A1 expression (FDR < 0.05, Fig. 3A). The top 50 positively and negatively correlated genes are shown in heatmaps (Fig. 3B–C). GO and KEGG enrichment analyses were conducted based on COL12A1-related genes ([13, 14]). At an adjusted p-value < 0.1, 221 biological processes (BP), 31 cellular components (CC), 24 molecular functions (MF), and 14 KEGG pathways were identified. Representative GO terms and KEGG pathways are shown in bubble plots, with three examples from each category (Fig. 3D–E). The results indicate that COL12A1 coexpression is mainly associated with extracellular structure organization and extracellular matrix organization. STRING network analysis (Fig. 3F) and circular plots (Fig. 3G–H) were used to further visualize these functional associations.

### Correlation between COL12A1 and methylation genes

**Fig. 4:**
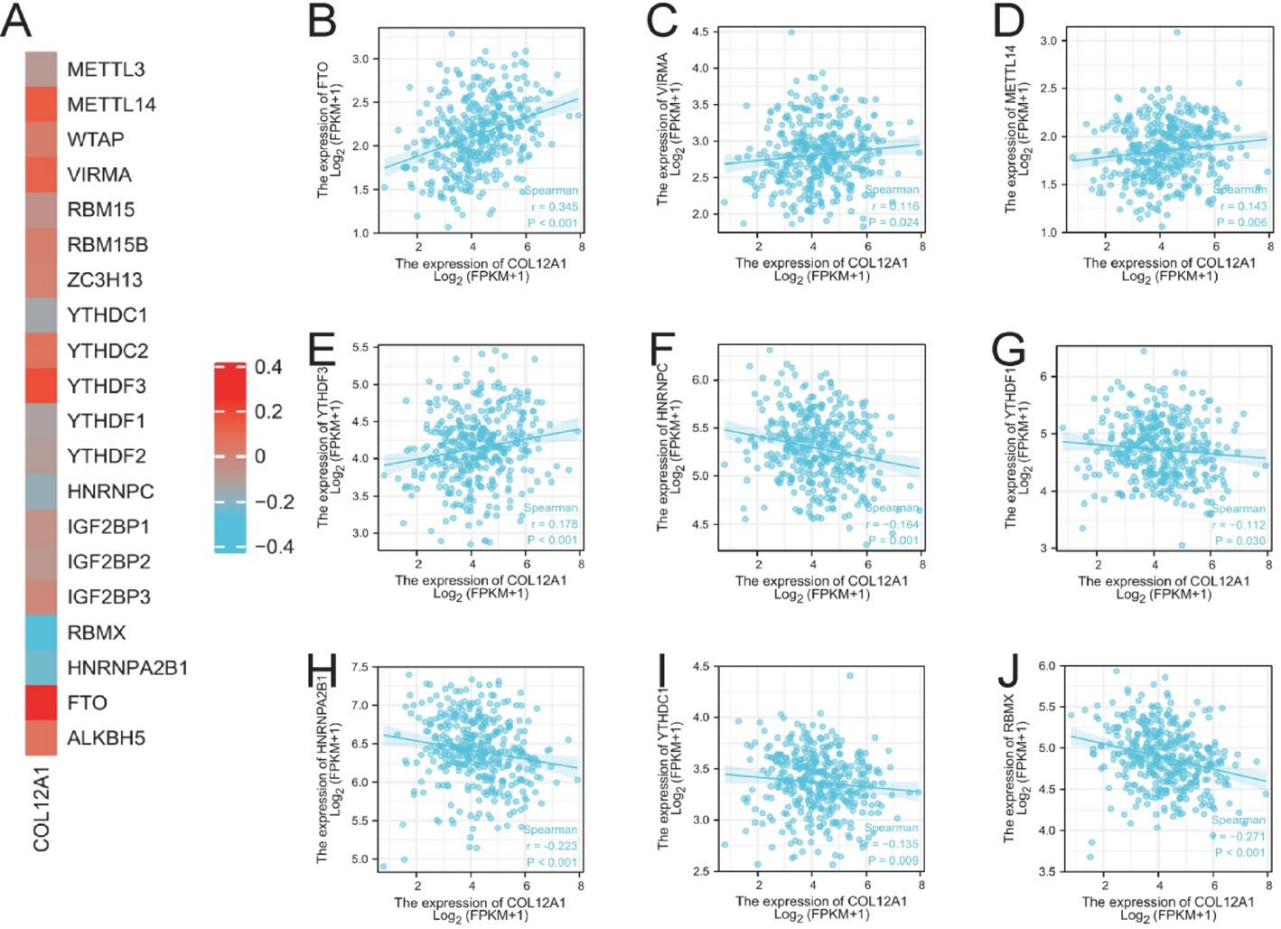
Relationship between methylation-associated genes and COL12A1 expression in STAD. (**A**) Heatmap of the correlation between COL12A1 expression and methylation genes. **B–J** Significant Spearman correlations between COL12A1 and FTO, VIRMA, METTL14, YTHDF3, HNRNPC, YTHDF1, HNRNPA2B1, YTHDC1, and RBMX in the TCGA database

The MethSurv database was used to examine COL12A1 DNA methylation levels and the prognostic value of various CpG sites [15]. A total of 41 methylated CpG sites were identified, with cg13319757, cg13395133, and cg16633701 showing the highest methylation levels (Figure S1). Among these, 17 CpG sites exhibited prognostically significant methylation levels: cg03503642, cg03564793, cg04504006, cg04611812, cg08009622, cg11353250, cg11526848, cg12488810, cg12801474, cg13319757, cg13395133, cg14375912, cg15089846, cg21112099, cg24897255, and cg26997327 (*p* < 0.05; Table 2). High methylation at these sites was significantly associated with poorer overall survival. To further explore methylation’s role, we analyzed the correlation between COL12A1 expression and 24 key methylation-related genes from TCGA, including ALKBH5, FTO, HNRNPA2B1, HNRNPC, IGF2BP1, IGF2BP2, IGF2BP3, METTL3, METTL14, RBMX, RBM15, RBM15B, VIRMA, WTAP, YTHDC1, YTHDC2, YTHDF1, YTHDF2, YTHDF3, and ZC3H13. The heatmap in Fig. 4A illustrates the relationships between COL12A1 expression and these methylation genes. Notably, COL12A1 expression correlated positively with FTO, VIRMA, METTL14, and YTHDF3, and negatively with HNRNPC, YTHDF1, HNRNPA2B1, YTHDC1, and RBMX (Fig. 4B–J). Among these, COL12A1 showed a strong positive correlation with FTO (*r* = 0.345, *p* < 0.001) and a strong negative correlation with RBMX (*r* = -0.271, *p* < 0.001). These findings indicate that COL12A1 is closely associated with methylation regulation.

**Table 2:** Impact of hypermethylation level on STAD prognosis

CpG	HR	*P*.value
5’UTR-N_Shore-cg03503642	1.645	0.019363893
3’UTR-Island-cg03564793	0.62	0.022793414
5’UTR-N_Shore-cg04504006	0.65	0.01383303
Body-Island-cg04611812	0.586	0.018738755
TSS1500-Island-cg08009622	0.579	0.014385012
3’UTR-Island-cg11353250	0.596	0.018416616
3’UTR-N_Shore-cg11526848	0.674	0.025113165
Body-Open_Sea-cg12488810	0.674	0.02529662
5’UTR-Island-cg12801474	0.467	0.000994599
Body-Open_Sea-cg13319757	0.707	0.035739547
Body-Open_Sea-cg13395133	0.645	0.037276974
5’UTR-Island-cg14375912	0.681	0.021136497
Body-S_Shelf-cg15089846	1.384	0.049335084
5’UTR-S_Shore-cg21112099	1.435	0.044691983
TSS200-Island-cg24897255	0.653	0.042656768
Body-Island-cg26997327	0.677	0.018542646

### COL12A1 is associated with the infiltration of immune cells within GC tissue

**Fig. 5:**
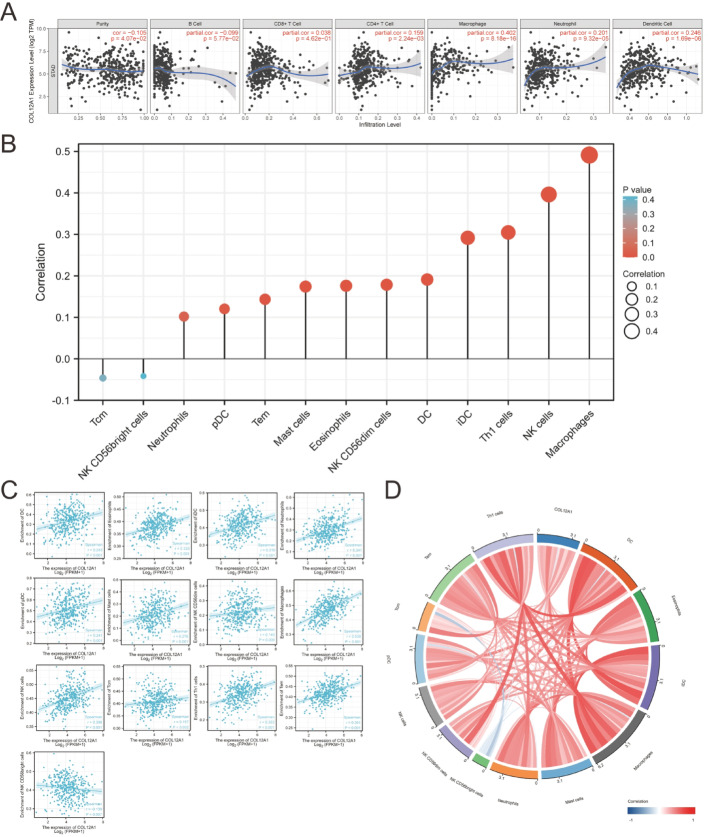
Relationship between immune infiltration and the expression of COL12A1 in STAD. (**A**) After purity regulation, the COL12A1 expression level was associated with the infiltration level of STAD in B cells, CD8 + T cells, CD4 + T cells, macrophages, neutrophils, and dendritic cells. (**B**) Correlations of 24 immune cell subtypes in the high and low COL12A1 expression groups in the STAD tumor samples. (**C**) Infiltration levels of various immune cells in STAD at different copy numbers (*p* < 0.01). (**D**) String diagram of the correlation of COL12A1 with specific immune cells in STAD (*p* < 0.01)

Immune infiltration plays a critical role in the tumor microenvironment. We utilized the TIMER database to assess immune cell infiltration in STAD tissues [16]. As shown in Fig. 5A, COL12A1 expression was significantly correlated with infiltration levels of macrophages, CD4 + T cells, dendritic cells (DCs), and neutrophils in gastric cancer tissues (*p* < 0.01). However, no significant correlation was observed with B cells or CD8 + T cells (*p* > 0.05). The correlation coefficient between COL12A1 and macrophage infiltration was notably high (*r* = 0.402). To further understand COL12A1’s role in immune regulation, the cohort was divided into high (188 samples) and low (187 samples) COL12A1 expression groups. We then compared the differential abundance of 24 immune cell types [17] between these groups (Fig. 5B). Immune cell types showing significant differences (*p* < 0.01) are presented in a lollipop plot, with their correlations visualized via chord diagrams. The proportions of macrophages (*r* = 0.520), effector memory T cells (Tem) (*r* = 0.409), natural killer (NK) cells (*r* = 0.388), Th1 cells (*r* = 0.367), and neutrophils (*r* = 0.360) were markedly different between the high and low COL12A1 groups (Fig. 5C–D). These results suggest that COL12A1 expression modulates immune cell infiltration in gastric cancer tissues.

### Single-cell analysis of COL12A1 across cancers

**Fig. 6:**
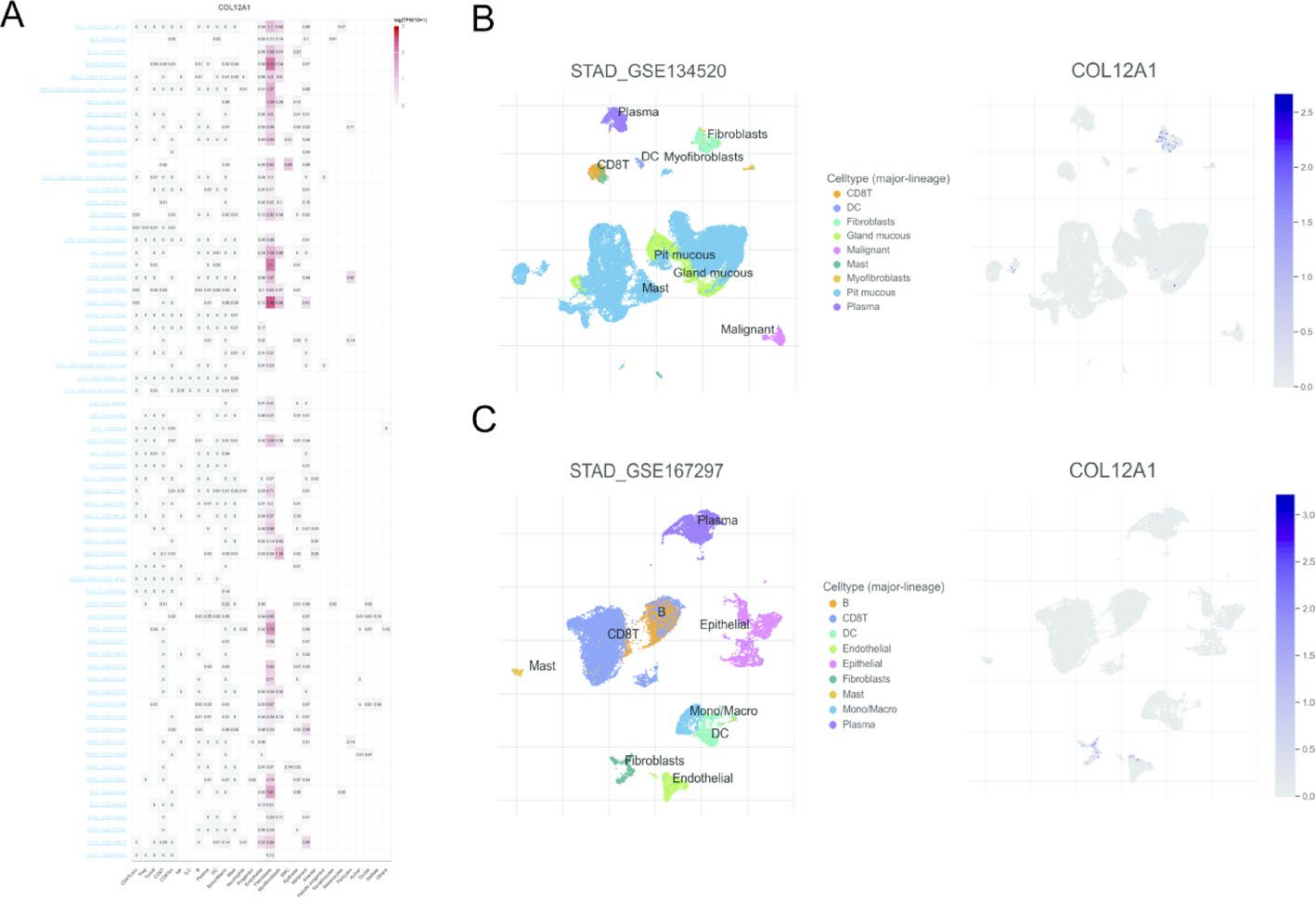
Single-cell analysis of COL12A1 across cancers. (**A**) Single-cell expression of COL12A1 across all the cancer tumor tissues. (**B**) Scatter plot showing the distribution of different cell types in the GSE134520 dataset. (**C**) Scatter plot showing the distribution of different cell types in the GSE167297 dataset

To explore the cellular origin of COL12A1-high-expressing cells in tumors, single-cell RNA sequencing data from 67 tumor samples were analyzed. A heatmap illustrating COL12A1 expression across 29 cell types in the tumor microenvironment is shown in Fig. 6A. The analysis revealed that COL12A1 was predominantly expressed in fibroblasts and myofibroblasts. Specifically, datasets GSE134520 and GSE167297, which include STAD patient data, confirmed significantly elevated COL12A1 expression in fibroblasts (Fig. 6B–C). These findings suggest that COL12A1 expression in fibroblasts may contribute to a more aggressive tumor phenotype via enhanced extracellular matrix remodeling and stromal-immune interactions.

### Drug sensitivity analysis

The response of cancer to drugs is a complex process influenced by multiple factors [18]. Previous studies indicate that genes highly associated with drug sensitivity often serve as oncogenes and direct targets of therapeutic agents [19]. Therefore, investigating genomic markers alongside anticancer drug sensitivity can identify new drug targets and key predictive biomarkers to enhance treatment efficacy [20]. RNAactDrug is a comprehensive resource integratingvarious databases to explore relationships between drug sensitivity and RNA molecular expression [21]. Using RNAactDrug, we analyzedthe correlation between COL12A1 mRNA expression and sensitivity to anticancer drugs across multiple histological subtypes. As shown in Table 3, palbociclib, geldanamycin, rifocin, eupachlorin, nanaomycin, and nilotinib were negatively correlated with COL12A1 mRNA levels. Conversely, vorinostat, zibotentan, amuvatinib, alectinib, and ruxolitinib showed positive correlations. Notably, alectinib, ruxolitinib, and cabozantinib exhibited significant positive correlations with COL12A1 mRNA expression. These results suggest that drugs such as palbociclib, geldanamycin, rifocin, eupachlorin, nanaomycin, and nilotinib may hold potential as therapeutic agents for gastric cancer.

**Table 3:** Correlations between anticancer drug sensitivity and COL12A1 mRNA

Compound	RNA type	RNA molecule	Omics	Source	Pearson	*P* ^*^	Spearman	*P* ^*^
Palbociclib	mRNA	COL12A1	Expression	CellMiner	-0.4952575	**	-0.551305391	**
geldanamicin	mRNA	COL12A1	Expression	CellMiner	-0.4723276	*	-0.483938201	**
rifocin	mRNA	COL12A1	Expression	CellMiner	-0.44642	**	-0.438146049	*
eupachlorin	mRNA	COL12A1	Expression	CellMiner	-0.42063804	*	-0.434882881	*
nanaomycin	mRNA	COL12A1	Expression	CellMiner	-0.4036568	*	-0.409203325	*
Nilotinib	mRNA	COL12A1	Expression	CellMiner	-0.3992817	*	-0.462416986	*
Vorinostat	mRNA	COL12A1	Expression	GDSC	0.2368774	****	0.278214577	****
Zibotentan	mRNA	COL12A1	Expression	GDSC	0.197256	****	0.225570257	****
Amuvatinib	mRNA	COL12A1	Expression	GDSC	0.22927364	****	0.275886865	****
Alectinib	mRNA	COL12A1	Expression	GDSC	0.14317301	****	0.211575182	****
Ruxolitinib	mRNA	COL12A1	Expression	GDSC	0.184881	****	0.243649687	****
Cabozantinib	mRNA	COL12A1	Expression	GDSC	0.09144408	*	0.110760869	**

### COL12A1 overexpression in GC

**Fig. 7:**
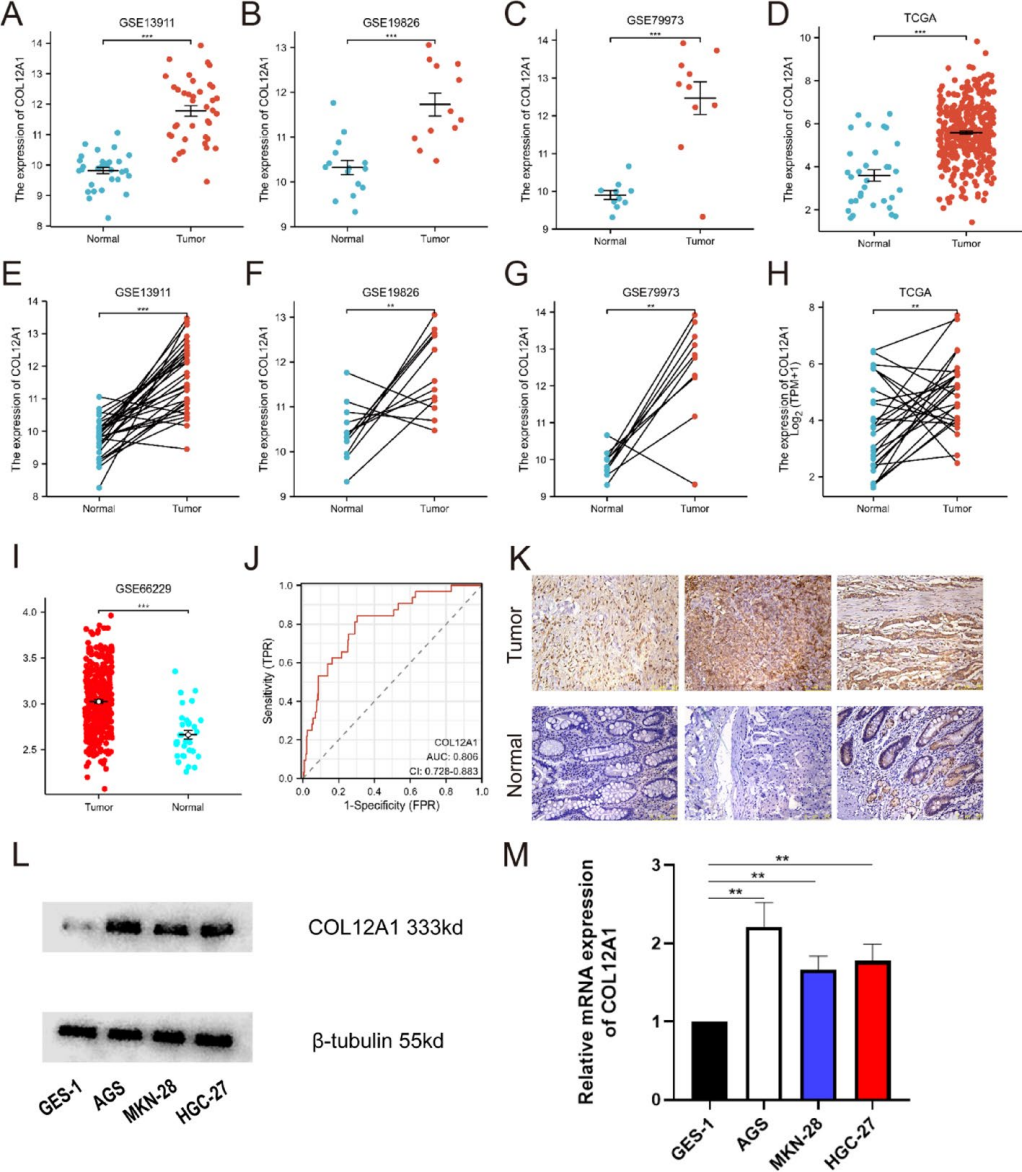
COL12A1 expression is upregulated in gastric cancer. (**A**–**D**) COL12A1 expression in tumors and unpaired paraneoplastic tissues in the Gene Expression Omnibus (GEO) datasets of the GSE13911, GSE19826, GSE79973, and TCGA datasets (**E–H**). Expression levels of COL12A1 in tumors and paired adjacent tissues in the GSE13911, GSE19826, GSE79973, and TCGA datasets of the GEO database. (**I**–**J**). External dataset GSE66229 (ACRG) validates COL12A1 expression and diagnostic value. (**K**) Validation of COL12A1 expression levels in GC using clinical samples (immunohistochemistry). (**L**–**M**) Western blot and qPCR was used to detect the levels of COL12A1 in different STAD cell lines

COL12A1 was significantly upregulated in gastric cancer tissues in the GSE13911, GSE19826, GSE79973 datasets, and TCGA database (*p* < 0.001; Fig. 7A–D). In all four datasets, COL12A1 expression was markedly higher in tumor samples compared to matched and unmatched normal tissues (Fig. 7E–H). Immunohistochemical analysis of patient specimens further confirmed elevated COL12A1 protein levels in tumor tissues relative to adjacent normal gastric tissues (Fig. 7I). Consistently, qRT‒PCR analysis demonstrated increased COL12A1 mRNA expression in tumor tissues compared to normal counterparts (Fig. 7J). Moreover, compared to normal epithelial cells, COL12A1 expression was significantly higher in the AGS and HGC-27 gastric cancer cell lines, with relative expression values of 1.000 ± 0.000 vs. 2.213 ± 0.308 (*P* = 0.0024) and 1.000 ± 0.000 vs. 1.665 ± 0.172 (*P* = 0.0026), respectively (Fig. 7K).

### Knockdown of COL12A1 suppresses the malignant phenotype of GC cells

**Fig. 8:**
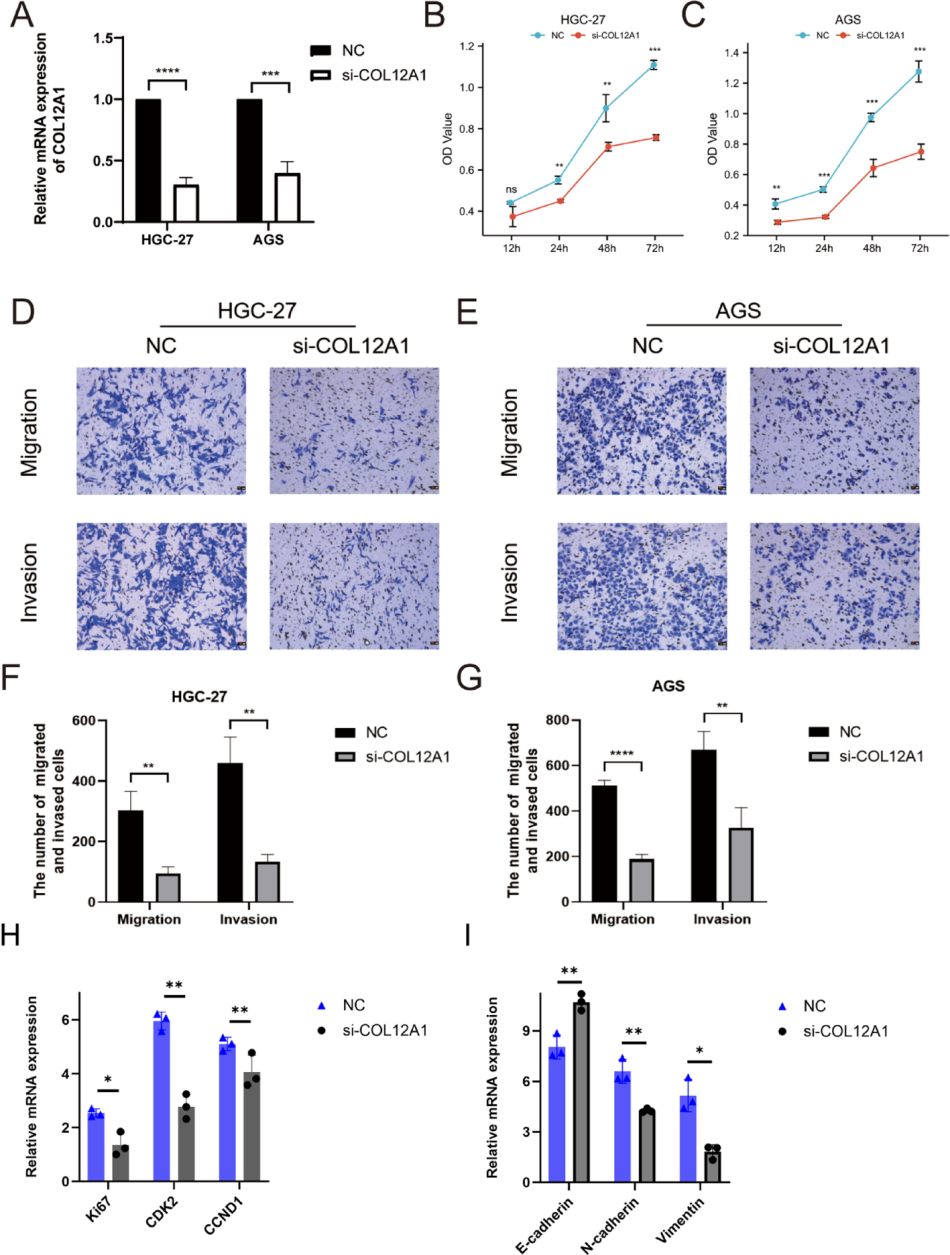
Efficacy of COL12A1 on gastric cancer progression. (**A**) PCR was used to verify the interference efficiency of COL12A1. (**B–C**) Effect on the proliferation of gastric cancer cells after COL12A1 gene expression was silenced. (**D–E**) Effects of COL12A1 knockdown on the migration and invasion of gastric cancer cells. (**F-G**) Statistical analysis of the migration and invasion of gastric cancer cells after the knockdown of COL12A1. (**H-I**) mRNA exprssion of Ki67, CDK2, CCND1, E-cadherin, N-cadherin and Vimentin between NC and si-COL12A1 group

To investigate the role of COL12A1 in gastric cancer (GC) oncogenesis and progression, we performed knockdown experiments in GC cell lines. HGC-27 and AGS cells were transfected with siRNA targeting COL12A1 (si-COL12A1) or a negative control (NC) using Lipo3000. As shown in Fig. 8A, si-COL12A1 significantly reduced COL12A1 expression in both cell lines (relative expression levels in HGC-27 cells: 1.000 ± 0.000 vs. 0.304 ± 0.059, *P* < 0.0001; in AGS cells: 1.000 ± 0.000 vs. 0.398 ± 0.095, *P* = 0.0004). CCK-8 assays revealed that, at 72 h, the absorbance values at 450 nm were significantly lower in the si-COL12A1 groups compared to NC, indicating inhibited cell proliferation (HGC-27: 1.109 ± 0.022 vs. 0.757 ± 0.014; AGS: 1.276 ± 0.069 vs. 0.750 ± 0.050; Fig. 8B and C). Furthermore, Transwell assays demonstrated that COL12A1 knockdown markedly suppressed both migration and invasion abilities in HGC-27 and AGS cells. Specifically, migrating and invading HGC-27 cells decreased from 302.0 ± 64.8 to 94.0 ± 23.1 (*P* = 0.0064) and from 460.3 ± 85.6 to 133.7 ± 24.0 (*P* = 0.0031), respectively. Similarly, migrating and invading AGS cells decreased from 512.3 ± 22.4 to 187.3 ± 22.0 (*P* < 0.0001) and from 669.0 ± 81.0 to 326.3 ± 88.9 (*P* = 0.0078), respectively (Figs. 8D–G). These findings suggest that COL12A1 promotes gastric cancer cell growth and metastatic potential in vitro. Additionally, we examined the mRNA expression of proliferation and epithelial–mesenchymal transition (EMT) markers in the NC and si-COL12A1 groups. Notably, the expression of proliferation markers Ki67, CDK2, and CCND1 was significantly reduced following COL12A1 knockdown, indicating decreased proliferative capacity. In terms of EMT markers, E-cadherin was upregulated, whereas N-cadherin and Vimentin were downregulated in the si-COL12A1 group, suggesting suppression of EMT upon COL12A1 silencing (Figs. 8H–I).

## Discussion

Gastric cancer (GC) is the fifth most common malignant tumor worldwide and is characterized by high aggressiveness, significantly impairing patients’ quality of life and health ( [22]– [23]). Due to limited patient awareness, variable diagnostic capabilities, and shortcomings of current screening biomarkers, most GC patients are diagnosed at intermediate or advanced stages, often presenting with lymph node involvement and distant metastases. This leads to poor clinical outcomes and prognosis [24]. Therefore, beyond raising awareness and promoting early screening, there is a pressing need to develop novel and more effective early diagnostic biomarkers for GC that hold theoretical, clinical, and socioeconomic value. In addition to genetic predispositions and chronic gastric conditions, immune factors also play a crucial role in the development and recurrence of GC [25].

Collagen is a key structural protein in the extracellular matrix (ECM) and the most abundant functional protein in the human body [26]. Collagen family members, including COL12A1, have been implicated in tumor proliferation and metastasis by remodeling the ECM and inducing epithelial-mesenchymal transition (EMT) ( [27]– [9]). Aberrant expression of COL12A1 has been observed in colorectal cancer, where it may regulate protein ubiquitination via the Wnt signaling pathway through interactions with transcription factors SOX9 and the kinase CSNK1A1L [28]. Li et al. demonstrated that COL12A1, positively regulated by METTL3, exerts oncogenic effects in esophageal squamous cell carcinoma (ESCC) progression [29]. Additionally, Chen et al. found a strong association between COL12A1 expression and overall survival (OS) in pancreatic cancer patients, with aberrant COL12A1 linked to multiple pathological processes [30]. Moreover, ovarian cancer cells with high COL12A1 expression exhibit increased resistance to antineoplastic drugs [31]. Collectively, these studies highlight the significance of COL12A1 in cancer, although the mechanisms underlying its role in tumor progression require further investigation.

Compared to normal tissues, COL12A1 expression is significantly elevated in GC samples and inversely correlated with OS. Further analysis confirmed a positive association between COL12A1 levels and tumor T stage as well as histological grade, suggesting its potential as a diagnostic marker for intermediate to advanced gastric adenocarcinoma (STAD). These findings support the clinical utility of COL12A1 in GC diagnosis. Moreover, COL12A1 suppression inhibited GC cell proliferation, migration, and invasion, confirming its role in promoting gastric tumor growth. Functional network analysis revealed that COL12A1 may contribute to STAD through pathways involved in extracellular structure and matrix organization. We also exploredthe relationship between COL12A1 expression, DNA methylation, prognostic CpG sites, and m6A-related regulators. Among 17 CpG sites analyzed, cg13319757, cg13395133, and cg16633701 exhibited the highest methylation levels. Patients with high COL12A1 methylation showed poorer OS compared to those with low methylation. Correlation analyses further revealed that COL12A1 upregulation was significantly associated with nine methylation-related genes (FTO, VIRMA, METTL14, YTHDF3, NRNPC, YTHDF1, HNRNPA2B1, YTHDC1, and RBMX), underscoring its potential as an early diagnostic and prognostic biomarker for GC.

Tumor immunotherapy has revitalized cancer treatment, with immune cells infiltrating the tumor microenvironment (TME) playing key roles in tumor progression regulation ( [32]– [33]). Using the ESTIMATE algorithm, we assessed the association between COL12A1 and immune infiltration, finding significant correlations with macrophages, B cells, CD4 + T cells, and dendritic cells via TIMER analysis. Further evaluation of immune cell marker genes demonstrated associations between COL12A1 and macrophages, effector memory T cells (Tems), natural killer (NK) cells, Th1 cells, and neutrophils. Studies indicate that COL12A1 promotes recruitment of immune cells such as T lymphocytes, macrophages, and dendritic cells, enhancing their migration towards tumor sites. Additionally, COL12A1 activates dendritic cells, facilitates T lymphocyte activation and differentiation, and suppresses immunosuppressive cell functions, thereby strengthening antitumor immune responses. Importantly, COL12A1 expression also influences GC patient responses to immunotherapy, with higher expression correlating with improved efficacy of PD-1/PD-L1 inhibitors. This may result from COL12A1’s role in enhancing immune cell infiltration and TME immune activity.

Given the link between COL12A1 and immune infiltration in GC, therapeutic strategies targeting COL12A1 are being explored. Approaches such as anti-COL12A1 antibodies, small-molecule inhibitors, and gene therapies hold promise for suppressing tumor growth, modulating the immune microenvironment, and enhancing immunotherapy outcomes.

Chemoresistance remains a major barrier to effective cancer treatment, limiting the selection and success of antitumor drugs. Assessing drug sensitivity in cancer cells offers valuable insights for guiding clinical therapy [34]. Moreover, therapeutic outcomes partially depend on tumor genetic alterations, making the discovery of predictive biomarkers essential ( [35]– [20]). Correlation analyses via the RNAactDrug database identified COL12A1 as associated with drug sensitivity, supporting its potential as a predictive biomarker for monitoring treatment responses. Drugs such as palbociclib, geldanamycin, rifocin, eupachlorin, nanaomycin, and nilotinib, linked to COL12A1, may serve as novel therapeutic options for GC.

This study has limitations. First, mechanistic insights were primarily derived from bioinformatics analyses without experimental validation in vivo or in vitro. Additionally, the precise role of COL12A1 within the tumor immune microenvironment requires further elucidation. Lastly, the relationship between COL12A1 expression and chemosensitivity in GC lacks experimental confirmation. Future studies employing functional assays in patient-derived xenografts or genetically manipulated cell lines could clarify the therapeutic potential of targeting COL12A1.

**Fig. 9:**
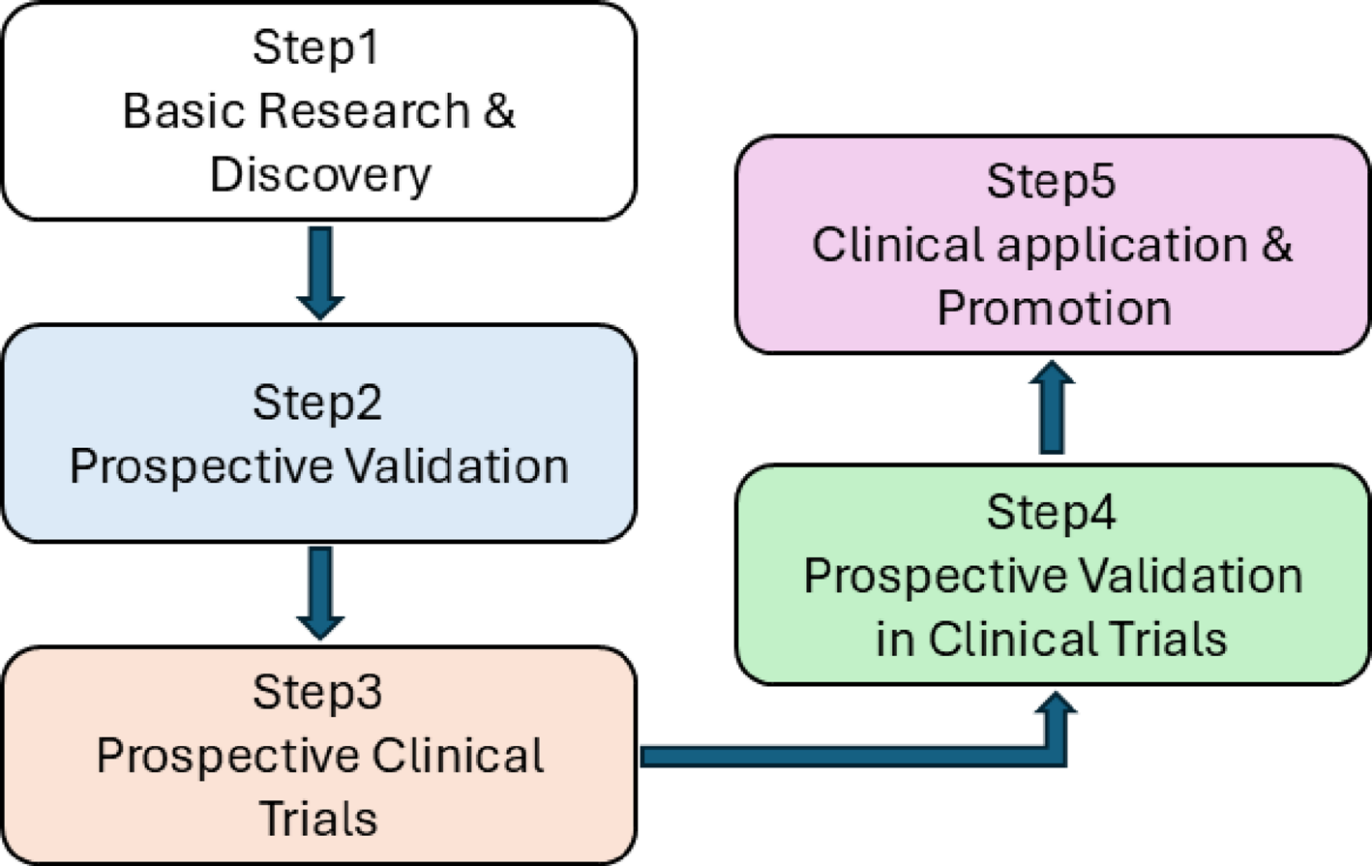
Biomarker translational pathway map for gastric cancer blockade therapy

To fully realize the promise of biomarkers like COL12A1 and PD-1 in gastric cancer, we need a robust translational path. The diagram illustrates precisely this biomarker translational process for gastric cancer patients receiving blockade therapy (Fig. 9). It covers five key stages: basic research and discovery, preclinical validation, retrospective clinical studies, prospective validation, and clinical application and promotion. The arrows clearly show the sequential progression from initial biomarker identification to final clinical utility. Integrating findings on biomarkers such as COL12A1 and PD-1 across these stages is crucial, as it highlights their vital role in predicting treatment response and guiding clinical decisions. This comprehensive approach is essential for moving from initial discoveries to validated clinical tools.

In conclusion, our findings support COL12A1 as a potential diagnostic biomarker and therapeutic target in GC. However, the molecular mechanisms by which COL12A1 regulates GC remain unclear and warrant further investigation. Moreover, evidence supporting COL12A1’s clinical utility is currently insufficient, necessitating additional comprehensive studies and clinical trials.

## Materials and methods

### GC patient dataset

The mRNA expression data (including 375 stomach adenocarcinoma [STAD] samples and 32 matched noncarcinoma tissue samples), clinical information, and survival data (TPM) from the TCGA database (https://cancergenome.nih.gov) were downloaded, and the data were log2(value + 1) processed. This work further analysed the levels of many GEO-derived genes (https://www.ncbi.nlm.nih.gov/geo/): GSE13911 (38 STAD samples and 31 paired adjacent nontumor samples), GSE19826 (15 gastric adenocarcinoma [STAD] samples and 12 matched adjacent nontumor samples), and GSE79973 (10 STAD samples and 10 paired normal nontumor samples). The R software limma package was used to identify differentially expressed genes (DEGs). The differential expression of COL12A1 was statistically analysed via the stats package as well as the car package in R software, and the data were visualized via the ggplot2 package. Additionally, COL12A1 protein expression was identified through immunohistochemistry (IHC) analysis.

### Survival analysis and creation of line graphs for predicting survival in patients with STAD

The RNA-seq data from the STAR process of the TCGA-STAD (gastric adenocarcinoma) project were downloaded from the TCGA database (https://portal.gdc.cancer.gov) and extracted in TPM format, and the data were log2(value + 1) processed. The 375 patients whose survival information was available were divided into high- and low-expression groups according to the median value of COL12A1 expression, and the proportional risk hypothesis test and fitted survival regression were performed via the survival package. The results were visualized via the survminer package as well as the ggplot2 package. COL12A1 levels in patients with GC and overall survival (OS) and disease-specific survival (DSS) were plotted via Kaplan–Meier curves. Univariate survival regression was also performed to calculate hazard ratios (HRs) and 95% confidence intervals. In this study, for the dichotomous endings (1 vs. 0, where the reference group is 0), the rms and resource selection packages in R software were used for processing. The data were first cleaned, then a binary logistic model was constructed via the glm function, and a nomogram correlation model was constructed and visualized via the rms package. The clinicopathological factors used to independently predict patient prognosis were selected via Cox regression, and a league table was constructed to assess the 1-, 3-, and 5-year OS probabilities in patients with STAD.

### Correlation of COL12A1 expression with clinical features

To analyse the clinical relevance of the COL12A1 gene in GC patients, we mined the clinical data of 375 patients from the TCGA project. Associations of COL12A1 levels with clinical parameters for GC patients were tested through chi-square analysis via the stats package in R software.

### Gene methylation analysis

We used the MethSurv database (https://biit.cs.ut.ee/methsurv/) to analyse DNA methylation sites in COL12A1 and the prognostic value of CpG methylation. Expression profile data for the methylated genes in TCGA were downloaded and correlated with COL12A1.

### Immunoinfiltration analysis

This work utilized the TIMER database (https://cistrome.shinyapps.io/timer/) to analyse the correlation of the COL12A1 level with the degree of infiltration of immune cells within STAD. The 24 immune cell types within the tumor were evaluated via single-sample gene set enrichment analysis (ssGSEA),and statistically significant (*p* < 0.01) results are listed. In addition, this work adopted Spearman’s test to determine the relationship between COL12A1 expression and the above immune cells.

### Single-cell analysis

Leveraging the Tumor Immune Single-Cell Hub (TISCH), we conducted a systematic analysis of pancancer single-cell transcriptomes to evaluate the expression profile of COL12A1 across different cell types. Heatmaps and scatter plots were generated to visualize the results (https://TISCH.comp-genomics.org/).

### Coexpression and enrichment analysis of COL12A1

To identify differentially expressed genes (DEGs) associated with COL12A1, we utilized the LinkedOmics database (http://www.linkedomics.org/login.php), which offers comprehensive multiomics data analysis tools for cancer research within and across various tumor types in The Cancer Genome Atlas (TCGA) database. Specifically, we focused on RNA-seq data from the TCGA-Stomach Adenocarcinoma (STAD) dataset for analysis. For the statistical analysis of DEGs coexpressed with COL12A1, Spearman’s test was used to assess the correlation between gene expression levels. DEGs were visualized via a heatmap and volcano plot. The heatmap provides a graphical representation of gene expression patterns, whereas the volcano plot displays the statistical significance and fold change of DEGs. The significance threshold for DEG selection was set at a false discovery rate (FDR) of less than 0.05.

### Drug sensitivity analysis

RNAactDrug (http://biobigdata.hrbmu.edu.cn/RNAactDrug) is a synthetically extensive database used in the analysis of drug sensitivity. RNAactDrug was used to predict which anticancer drugs correlated with COL12A1 via Pearson correlation analysis, with a significance of *P* < 0.05.

### Cell culture and transfection

Healthy gastric cells (GES-1) and human GC cells (AGS, HGC-27) were obtained from Shanghai Zhongqiao Xinzhou Biotechnology Co. The cells were cultivated in 1640 medium (Gibco, USA) and DMEM (HyClone, USA); the medium contained 10% fetal bovine serum (Ausgenex, Australia), followed by growth to approximately 50% confluence in six-well plates (NEST Biotechnology Co., Wuxi, China) 1 day before transfection. We transfected siRNA sequences (GenePharma, Shanghai, China) with Lipofectamine 3000 (Thermo Fisher, CA, USA). The medium of the cells was changed to 10% FBS medium 6 h after transfection, and the cells were cultured for a 24–72-h period. The COL12A1 siRNA sequences used for the HGC-27 and AGS cell lines were as follows: siRNA-COL12A1 (5’-GCUGAUGAAGUCGAAUUAATT-3’, 3’- UUAAUUCGACUUCAUCAGCTT-5’) [9].

### Quantitative reverse transcription polymerase chain reaction (qRT‒PCR)

Total RNA was extracted from GES-1, MKN-28, HGC-27, and AGS cells by lysing the cells with TRIzol (Takara, Japan), and a Primer Script RT Kit (Takara, Japan) was used for reverse transcription. This work used SYBR Green qPCR Mix (PROTEINBIO, China) for qRT‒PCR with the following primers: COL12A1, 5’-CAACGGATGGGCCTACTAAA-3’ (forward, F), 5’-TCCACTGGCTTTGTCGAAC-3’ (reverse, R); GAPDH, 5’-TCGGAGTCAACGGATTTGGT-3’ (F), 5’-TTCCCGTTCTCAGCCTTGAC-3’ (R). Relative COL12A1 expression was normalized to that of GAPDH and the negative control, and expression alterations were evaluated via the 2^−ΔΔCT^ method [10]. Other primes are listed in Table S1.

### Western blot assay

Total protein was extracted from GES-1, MKN-28, HGC-27, and AGS cells using RIPA lysis buffer (Beyotime, China) supplemented with protease and phosphatase inhibitors. Protein concentrations were determined using a BCA Protein Assay Kit (Beyotime, China). Equal amounts of protein (30–50 µg) were separated by 8–10% SDS-PAGE and transferred onto PVDF membranes (Millipore, USA). Membranes were blocked with 5% non-fat milk in TBST for 1 h at room temperature, then incubated overnight at 4 °C with rabbit primary antibodies against COL12A1 (1:1000, ab121304, Abcam, UK) and β-tubulin (1:2000, ab6046, Abcam, UK) as a loading control. After washing, membranes were incubated with HRP-conjugated anti-rabbit secondary antibody (1:5000, ab6721, Abcam, UK) for 1 h at room temperature. Protein bands were visualized using an enhanced chemiluminescence (ECL) detection kit (Thermo Fisher Scientific, USA).

### Immunohistochemistry (IHC) analysis

Gastric cancer (GC) tissues and adjacent normal tissues were first fixed in 10% formalin and subsequently embedded in paraffin to create tissue microarrays. The tissue sections were then treated with specific primary antibodies. After antigen retrieval, the sections were blocked at room temperature with 1% BSA (Thermo Fisher, CA, USA) in TBST buffer for 2 h. The sections were incubated overnight at 4 °C with primary antibodies. After three washes, the sections were incubated at room temperature with HRP polymer-conjugated secondary antibodies (Thermo Fisher, CA, USA). The staining was developed using 3,3’-diaminobenzidine (DAB) substrate, followed by counterstaining with hematoxylin for contrast enhancement.

### Cell proliferation analysis

Cell proliferation was assessed via the Cell Counting Kit-8 (CCK-8) assay (APExBIO, USA) according to the manufacturer’s instructions. Briefly, HGC-27 and AGS gastric cancer cells (3,000 cells/well) were inoculated into 96-well plates 24 h after transfection with either COL12A1 siRNA or negative control (NC) siRNA. To monitor cell growth, 10 µL of CCK-8 solution was added to each well at designated time points: 12 h, 24 h, 48 h, and 72 h of culture. Following the addition of CCK-8, the cells were further incubated for 2 h at 37 °C in a humidified incubator with 5% CO2. The absorbance of the culture medium at 450 nm was then measured via a Tecan plate reader (Tecan, Switzerland). Higher absorbance values correspond to greater numbers of viable cells and, therefore, higher proliferation rates.

### Transwell assays

The upper cell chamber was lined with matrix gel and placed in an incubator to promote coagulation (100 µL, matrix collagen solution:1640 vacuole = 1:20) 4–6 h in advance. After transfection, the cells were resuspended in 100 µL of basal medium and incubated in the top chamber (Corning Inc., USA), followed by the addition of 650 µL of complete medium to the basolateral chamber. Next, methanol was added to immobilize the cells under the membrane, followed by 5% crystal violet staining.

### Statistical analysis

R (version 3.6.3), Adobe Illustrator 2020 and GraphPad Prism 8 (version 8.0.2) were used for graph production and statistical analysis. Diverse groups were examined for significance through a t test (*p* < 0.05). In all analyses, *, **, and *** denote *p* < 0.05, *p* < 0.01, and *p* < 0.001, respectively.

## Conclusions

COL12A1 is overexpressed in GC and has clinical relevance. Additionally, COL12A1 promoted GC cell growth, invasion and migration. Gene mutation, methylation, and immune infiltration possibly explain the oncogenic effect of COL12A1 on GC. Collectively, our findings provide valuable clues for the development of novel diagnostic and therapeutic approaches for the treatment of GC.

## Supplementary Information

Below is the link to the electronic supplementary material.

Supplementary Material 1.

Supplementary Material 1.

## Data Availability

The datasets generated and/or analysed during the current study are available in the Figshare repository (DOI: 10.6084/m9.figshare.29815130). Publicly available datasets were also obtained from established repositories such as GEO, TCGA, and LinkedOmics. Additional analyses were performed using online tools and databases, including TIMER and CIBERSORT for immune infiltration analysis, TISCH for single-cell expression analysis, MethSurv for DNA methylation and prognostic CpG site analysis, GDSC for drug sensitivity prediction, and RNAactDrug for drug–gene interaction profiling.

## Abbreviations

COL12A1: Collagen type XII alpha 1 chain
GC: Gastric cancer
IHC: Immunohistochemistry
ECM: Extracellular matrix
TCGA: The Cancer Genome Atlas
co-DEGs: Coexpressed differential genes
GEO: Gene Expression Omnibus
OS: Overall survival
DFS: Disease-free survival (DFS)
GO: Gene Ontology
KEGG: Kyoto Encyclopedia of Genes and Genomes
TIMER: Tumor immune estimation receptor (TIMER)
TME: Tumor microenvironment (TME)
FACIT: Fibril-associated collagens
HR: Hazard ratio (HR)
STAD: Stomach adenocarcinoma
COSMI: Catalogue of Somatic Mutations in Cancer
ssGSEA: Single-sample gene set enrichment analysis (ssGSEA)
FDR: False discovery rate
CCK-8: Cell Counting Kit-8
siRNA: small interfering RNA
qRT‒PCR: Quantitative reverse transcription polymerase chain reaction
NC: Negative control
DAB: Diaminobenzidine
BP: Biological processes
CC: Cellular component
MF: Molecular function
DCs: Dendritic cells
NK: Natural killer
Tem: Transverse electromagnetic
Th1: T helper 1
ESCC: Esophageal squamous cell carcinoma

## References

[1] Smyth EC, Nilsson M, Grabsch HI, van Grieken NC, Lordick F. Gastric cancer. Lancet. 2020;396(10251):635–48. 10.1016/S0140-6736(20)31288-5. 32861308 10.1016/S0140-6736(20)31288-5

[2] Ajani JA, D’Amico TA, Bentrem DJ, Chao J, Cooke D, Corvera C, et al. Gastric cancer, version 2.2022, NCCN clinical practice guidelines in oncology. J Natl Compr Canc Netw. 2022;20(2):167–92. 10.6004/jnccn.2022.0008. 35130500 10.6004/jnccn.2022.0008

[3] Joshi SS, Badgwell BD. Current treatment and recent progress in gastric cancer. CA Cancer J Clin. 2021;71(3):264–79. 10.3322/caac.21657. 33592120 10.3322/caac.21657 PMC9927927

[4] Li Y, Feng A, Zheng S, Chen C, Lyu J. Recent estimates and predictions of 5-Year survival in patients with gastric cancer: A Model-Based period analysis. Cancer Control. 2022;29:10732748221099227. 10.1177/10732748221099227. 35499497 10.1177/10732748221099227 PMC9067041

[5] Xu S, Xu H, Wang W, Li S, Li H, Li T, et al. The role of collagen in cancer: from bench to bedside. J Transl Med. 2019;17(1):309. 10.1186/s12967-019-2058-1. 31521169 10.1186/s12967-019-2058-1 PMC6744664

[6] Han S, Wang Z, Liu J, Wang HD, Yuan Q. MiR-29a-3p-Dependent COL3A1 and COL5A1 expression reduction assists Sulforaphane to inhibit gastric cancer progression. Biochem Pharmacol. 2021;188:114539. 10.1016/j.bcp.2021.114539. 33819468 10.1016/j.bcp.2021.114539

[7] Cui X, Shan T, Qiao L. Collagen type IV alpha 1 (COL4A1) silence hampers the invasion, migration and Epithelial–Mesenchymal transition (EMT) of gastric cancer cells through blocking Hedgehog signalling pathway. Bioengineered. 2022;13(4):8972–81. 10.1080/21655979.2022.2053799. 35297303 10.1080/21655979.2022.2053799 PMC9161915

[8] Nallanthighal S, Heiserman JP, Cheon DJ. Collagen type XI alpha 1 (COL11A1): A novel biomarker and a key player in cancer. Cancers (Basel). 2021;13(5):935. 10.3390/cancers13050935. 33668097 10.3390/cancers13050935 PMC7956367

[9] Xiang Z, Li J, Song S, Wang J, Cai W, Hu W, et al. A positive feedback between IDO1 metabolite and COL12A1 via MAPK pathway to promote gastric cancer metastasis. J Exp Clin Cancer Res. 2019;38(1):314. 10.1186/s13046-019-1318-5. 31315643 10.1186/s13046-019-1318-5 PMC6637527

[10] Harshitha R, Arunraj DR, Real-time Quantitative PCR. A tool for absolute and relative quantification. Biochem Mol Biol Educ. 2021;49(5):800–12. 10.1002/bmb.21552. 34132460 10.1002/bmb.21552

[11] Park SY, Nomogram. An analogue tool to deliver digital knowledge. J Thorac Cardiovasc Surg. 2018;155(4):1793. 10.1016/j.jtcvs.2017.12.107. 29370910 10.1016/j.jtcvs.2017.12.107

[12] Vasaikar SV, Straub P, Wang J, Zhang B. LinkedOmics:Analysing multiomics data within and across 32 cancer types. Nucleic Acids Res. 2018;46(D1):D956–63. 10.1093/nar/gkx1090. 29136207 10.1093/nar/gkx1090 PMC5753188

[13] Xing Z, Chu C, Chen L, Kong X. The use of Gene Ontology terms and KEGG pathways for analysis and prediction of oncogenes. Biochim Biophys Acta. 2016;1860(11 Pt B):2725–34. 10.1016/j.bbagen.2016.01.012 26801878 10.1016/j.bbagen.2016.01.012

[14] Chen L, Zhang YH, Wang S, Zhang Y, Huang T, Cai YD. Prediction and analysis of essential genes using the enrichments of gene ontology and KEGG pathways. PLoS One. 2017;12(9):e0184129. 10.1371/journal.pone.0184129 28873455 10.1371/journal.pone.0184129 PMC5584762

[15] Modhukur V, Iljasenko T, Metsalu T, Lokk K, Laisk-Podar T, Vilo J, MethSurv. Epigenomics. 2018;10(3):277–88. 10.2217/epi-2017-0118. A Web Tool to Perform Multivariable Survival Analysis Using DNA Methylation Data. 10.2217/epi-2017-0118 29264942

[16] Li B, Li T, Liu JS, Liu XS. Computational Deconvolution of tumor-Infiltrating immune components with bulk tumor gene expression data. Methods Mol Biol. 2020;2120:249–62. 10.1007/978-1-0716-0327-7_18. 32124325 10.1007/978-1-0716-0327-7_18

[17] Bindea G, Mlecnik B, Tosolini M, Kirilovsky A, Waldner M, Obenauf AC, et al. Spatiotemporal dynamics of intratumoral immune cells reveal the immune landscape in human cancer. Immunity. 2013;39(4):782–95. 10.1016/j.immuni.2013.10.003. 24138885 10.1016/j.immuni.2013.10.003

[18] Liu X, Yang J, Zhang Y, Fang Y, Wang F, Wang J, et al. A systematicstudy on Drug-Response associated genes using baseline gene expressions of the cancer cell line encyclopedia. Sci Rep. 2016;6:22811. 10.1038/srep22811. 26960563 10.1038/srep22811 PMC4785360

[19] Szakács G, Annereau JP, Lababidi S, Shankavaram U, Arciello A, Bussey KJ, et al. Predicting drug sensitivity and resistance: profiling ABC transporter genes in cancer cells. Cancer Cell. 2004;6(2):129–37. 10.1016/j.ccr.2004.06.026. 15324696 10.1016/j.ccr.2004.06.026

[20] Gonzalez de Castro D, Clarke PA, Al-Lazikani B, Workman P. Personalized cancer medicine: molecular diagnostics, predictive biomarkers, and drug resistance. Clin Pharmacol Ther. 2013;93(3):252–9. 10.1038/clpt.2012.237. 23361103 10.1038/clpt.2012.237 PMC3577635

[21] Dong Q, Li F, Xu Y, Xiao J, Xu Y, Shang D, et al. RNAactDrug: A comprehensive database of RNAs associated with drug sensitivity from Multi-Omics data. Brief Bioinform. 2020;21(6):2167–74. 10.1093/bib/bbz142. 31799597 10.1093/bib/bbz142

[22] Johnston FM, Beckman M. Updates on management of gastric cancer. Curr Oncol Rep. 2019;21(8):67. 10.1007/s11912-019-0820-4. 31236716 10.1007/s11912-019-0820-4

[23] Gullo I, Grillo F, Mastracci L, Vanoli A, Carneiro F, Saragoni L, et al. Precancerous lesions of the stomach, gastric cancer and hereditary gastric cancer syndromes. Pathologica. 2020;112(3):166–85. 10.32074/1591-951X-166. 33179620 10.32074/1591-951X-166 PMC7931572

[24] Sexton RE, Al Hallak MN, Diab M, Azmi AS. Gastric cancer: A comprehensive review of current and future treatment strategies. Cancer Metastasis Rev. 2020;39(4):1179–203. 10.1007/s10555-020-09925-3. 32894370 10.1007/s10555-020-09925-3 PMC7680370

[25] Krawczyk N, de Souza Espíndola Santos A, Lima J, Meyer A. Revisiting cancer 15 years later: exploring mortality among agricultural and nonagricultural workers in the Serrana region of Rio de Janeiro. Am J Ind Med. 2017;60(1):77–86. 10.1002/ajim.22660. 27699817 10.1002/ajim.22660 PMC6528178

[26] Ricard-Blum S. The collagen family. Cold Spring Harb Perspect Biol. 2011;3(1):a004978. 10.1101/cshperspect.a004978. 21421911 10.1101/cshperspect.a004978 PMC3003457

[27] Wang F, Zhang M. Circ_001209 aggravates diabetic retinal vascular dysfunction through regulating miR-15b-5p/COL12A1. J Transl Med. 2021;19(1):294. 10.1186/s12967-021-02949-5. 34233716 10.1186/s12967-021-02949-5 PMC8265106

[28] Wu Y, Xu Y. Integrated bioinformatics analysis of expression and gene regulation network of COL12A1 in colorectal cancer. Cancer Med. 2020;9(13):4743–55. 10.1002/cam4.2899. 32356618 10.1002/cam4.2899 PMC7333847

[29] Li J, Li Z, Xu Y, Huang C, Shan B. METTL3 facilitates tumor progression by COL12A1/MAPK signalling pathway in esophageal squamous cell carcinoma. J Cancer. 2022;13(6):1972–84. 10.7150/jca.66830. 35399719 10.7150/jca.66830 PMC8990406

[30] Chen S, Gao C, Yu T, Qu Y, Xiao GG, Huang Z. Bioinformatics analysis of a prognostic MiRNA signature and potential key genes in pancreatic cancer. Front Oncol. 2021;11:641289. 10.3389/fonc.2021.641289. 34094925 10.3389/fonc.2021.641289 PMC8174116

[31] Januchowski R, Świerczewska M, Sterzyńska K, Wojtowicz K, Nowicki M, Zabel M. Increased expression of several collagen genes is associated with drug resistance in ovarian cancer cell lines. J Cancer. 2016;7(10):1295–310. 10.7150/jca.15371. 27390605 10.7150/jca.15371 PMC4934038

[32] Zhang Y, Zhang Z. The history and advances in cancer immunotherapy: Understanding the characteristics of Tumor-Infiltrating immune cells and their therapeutic implications. Cell Mol Immunol. 2020;17(8):807–21. 10.1038/s41423-020-0488-6. 32612154 10.1038/s41423-020-0488-6 PMC7395159

[33] Kruger S, Ilmer M, Kobold S, Cadilha BL, Endres S, Ormanns S, et al. Advances in cancer immunotherapy 2019-Latest trends. J Exp Clin Cancer Res. 2019;38(1):268. 10.1186/s13046-019-1266-0. 31217020 10.1186/s13046-019-1266-0 PMC6585101

[34] Li Y, Umbach DM, Krahn JM, Shats I, Li X, Li L. Predicting tumor response to drugs based on Gene-Expression biomarkers of sensitivity learned from cancer cell lines. BMC Genomics. 2021;22(1):272. 10.1186/s12864-021-07581-7. 33858332 10.1186/s12864-021-07581-7 PMC8048084

[35] Wu Q, Yang Z, Nie Y, Shi Y, Fan D. Multi-Drug resistance in cancer chemotherapeutics: mechanisms and lab approaches. Cancer Lett. 2014;347(2):159–66. 10.1016/j.canlet.2014.03.013. 24657660 10.1016/j.canlet.2014.03.013

