# Supplementary material for: Multiomics analysis of COL12A1 as a promising prognostic biomarker for immune-related treatment of gastric cancer
Source: Discov Oncol. 2025 Oct 14;16:1876. doi: 10.1007/s12672-025-03405-2 (PMC12521706; doi:10.1007/s12672-025-03405-2)
Supplement: Supplementary file 1 — Supplementary Material 1. [file 12672_2025_3405_MOESM1_ESM.docx]

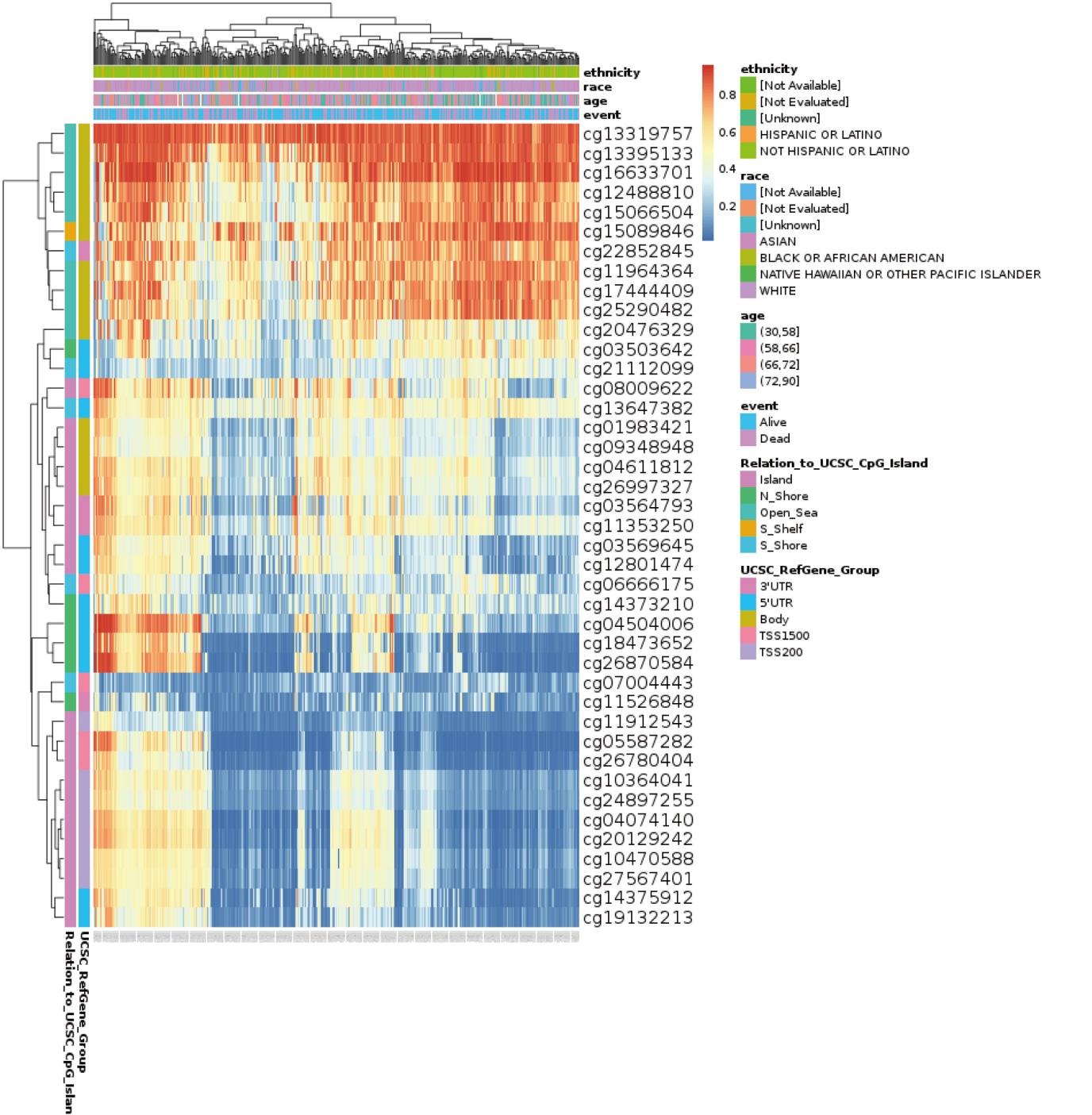


Supplementary Figure S1. COL12A1 methylation in STAD patients.

Supplementary Table S1. Primer sequences used for qRT-PCR analysis.

| qPCR | Forward Primer | Reverse Primer |
| --- | --- | --- |
| Ki67 | TCCTTTGGTGGGCACCTAAGACCTG | TGATGGTTGAGGTCGTTCCTTGATG |
| CDK2 | GCTTTTGGAGTCCCTGTTCG | GGTCCCCAGAGTCCGAAAGA |
| CCND1 (Cyclin D1) | CTGATTGGACAGGCATGGGT | GTGCCTGGAAGTCAACGGTA |
| E-cadherin (CDH1) | TACTATGATGAAGAAGGAGG | CGGAACCGCTTCCTTCATAG |
| N-cadherin (CDH2) | CAGTGGCCACCTACAAAG | AAATGAAACCGGGCTATC |
| Vimentin (VIM) | TGTCCAAATCGATGTGGATGTTTC | TTGTACCATTCTTCTGCCTCCTG |
