# Supplementary figures and images for: Multiomics analysis of COL12A1 as a promising prognostic biomarker for immune-related treatment of gastric cancer
Source: Discov Oncol. 2025 Oct 14;16:1876. doi: 10.1007/s12672-025-03405-2 (PMC12521706; doi:10.1007/s12672-025-03405-2)

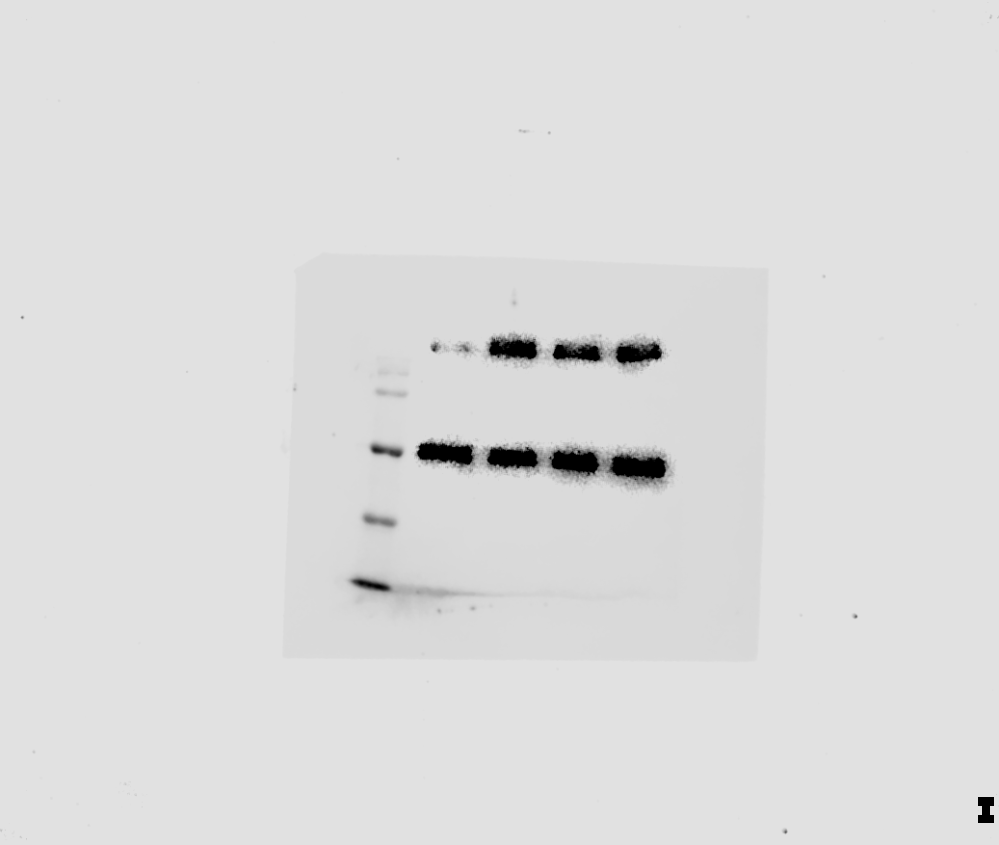

Supplement: Supplementary file 2 — Supplementary Material 1. [file 12672_2025_3405_MOESM2_ESM.png]
